# Data of the design of solar assisted district cooling systems

**DOI:** 10.1016/j.dib.2020.105541

**Published:** 2020-04-11

**Authors:** Dana Alghool, Tarek Elmekkawy, Mohamed Haouari, Adel Elomri

**Affiliations:** a Department of Mechanical and Industrial Engineering, Faculty of Engineering, Qatar University, Qatar; b Engineering Management and Decision Science, College of Science and Engineering, Hamad Bin Khalifa University, Qatar

**Keywords:** Solar assisted cooling system, Solar collectors, Absorption chillers, Annual cooling demand

## Abstract

The collected datasets are relevant and related to Optimization of Design and Operation of Solar Assisted District Cooling Systems [1] paper. Part of the data is collected on the main and common components of the system. That includes solar collectors unit price ($/m^2^), type, and efficiency; absorption chiller capacity (kW), type, initial cost ($), and COP; the hot/chilled water thermal energy storage tank type, initial cost ($) and capacity (kWh); and auxiliary boiler initial cost ($), capacity (kW), type and efficiency. The other part of the data is collected on hourly cooling demand over the year for the state of Qatar (kW), hourly global solar irradiance over the year for the state of Qatar (W/m^2^) and variable cost of producing and storing chilled and hot water ($/kWh, $/kW). The data are collected from different resources such as government websites, commercial websites, government sectors, journals and real-life case studies. The value of this data comes from that most of the data required to conduct such research in this area are available in one resource. Also, some of the data such as the annual hourly cooling demand and global solar radiation are not available online. Moreover, the collected data are already filtered and the units are consistent and ready to be used. Finally, the data considered to be crucial and the core of such research are available in this paper.

Specifications table

**Table:** 

Subject	Energy Engineering and Power Technology
Specific subject area	District Cooling Systems Coupled with Solar Energy
Type of data	Tables Graph Figure
How data were acquired	Derived Data, Commercial Websites, Governmental Website, Journal Papers, and Real-Life Case Studies.
Data format	Raw Analysed Filtered Derived
Parameters for data collection	The data of the annual hourly cooling demand and global solar radiations are collected on the year 2016 for state of Qatar. However, the rest of data are collected over the last ten years. The keywords used during the data collected are related to the system components such solar collectors, absorption chillers, auxiliary boilers, and hot and chilled water thermal energy storage (TES) tank
Description of data collection	Most of the data are collected from different sources such as commercial and governmental websites, real-life case studies and journal papers. However, the data of the global solar radiation is obtained from a government official. And the data of the annual cooling demand is derived from available data online using a certain approach.
Data source location	Department of Mechanical and Industrial Engineering, Faculty of Engineering, Qatar University, Qatar - Doha
Data accessibility	The complete datasets described in this study are available in the Mendeley Data repository. The reserved DOI is 10.17632/754wphy77k.1
Related research article	Dana Alghool, Tarek Elmekkawy, Mohamed Haouari, Adel Elomari, Optimization of design and operation of solar assisted district cooling systems, Energy Conversion and Management: X, 10.1016/j.ecmx.2019.100028

## 1 Value of the data

• Most of the collected data on the parameters of the model such as the annual hourly cooling demand and the annual hourly global solar radiation for the state of Qatar is not available on any sources. These types of data are considered to be essential and the core of any research conducted in this area. Hence, having easy access to this data would save a lot of time on the researcher.
• The collected data will open doors to other researchers by encouraging them to conduct researches in this area. Most of the data that could be used in this area of research is already available in this paper.
• Gaining access to such data would make it convenient for the researcher to conduct researches in this area, for instance, the cooling demand data represents the core of any research carried in this area. The researcher can scale down or up the generated cooling demand as per his requirement and experiment since the pattern of the demand would remain the same.
• The collected data combines all types of data such as capacity, fixed costs, variable costs, etc. required for each component of the system. The complete data of each component are collected from various sources. Hence, having all this data in one source would provide easy access to other researchers in the future.
• The collected data are filtered and ready for use. Most of the data collected on different parameters were collected and obtained from various sources, so most of the parameters had different measurements units such as capacity and currency units.

## 1 Data description

**Fig. 1:**
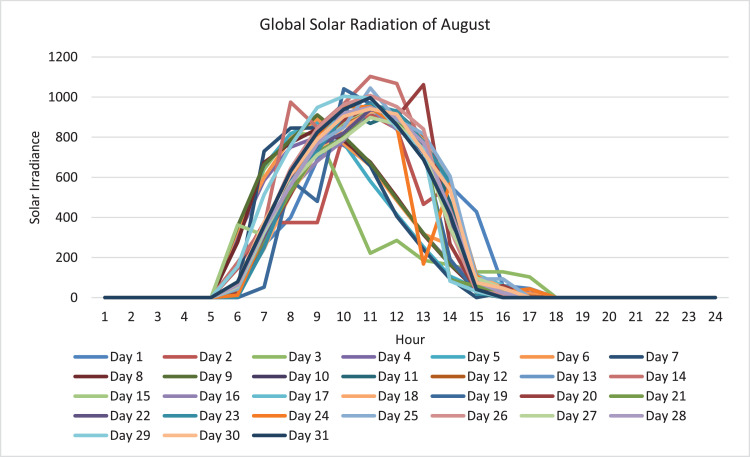
August global solar irradiance.

The data collected and presented in this paper are based on the parameters of the mathematical model developed in paper [1] which are related to the design and operation of solar assisted district cooling system. The data in this paper are presented in two forms as tables and figures. The figures are generated to represent the tabulated data conveniently and to make a better conclusion. The dataset included in the Mendeley Data repository is an excel sheet that has the data collected on the mathematical model parameters. The sheets of the excel file represent the data collected or derived on a certain parameter. The entire sets of the data collected on the parameters are presented in tables and two out of these parameters are also presented in figures for better data visibility. The type of collected data is related to, the annual hourly cooling demand for the state of Qatar (kW), where Fig. 3 shows the cooling demand of August as a sample and the complete data and figures are included in the repository; the annual hourly global solar radiation for the state of Qatar (W/m^2^), where Fig. 1 shows the global solar radiation of August as a sample and the complete data and figures are included in the repository; and the annual hourly variable cost of producing and storing chilled and hot water for the state of Qatar ($/kW and $/kWh) is obtained from [32]. Moreover, data specific to the components of the solar assisted district cooling system is collected. One of the components is the absorption chiller where data related to fixed cost ($), capacity (kW), and COP are collected. The collected data are shown in Table 1. The other component is solar collectors, where data related to the type, efficiency and fixed cost ($/m2) are collected. The collected data are shown in Table 2. other components are the hot and chilled water thermal energy storage (TES) tanks, where data related to the type of TES, capacity (kWh) and fixed cost of TES ($) are collected. The collected data are shown in Table 3. Finally, data on the auxiliary boiler component related to fixed cost ($), efficiency, and capacity (kW) are collected. The collected data are shown in Table 4. Hence, data on the five main components of the system were collected. The data collected on each parameter are explained below.

**Fig. 2:**
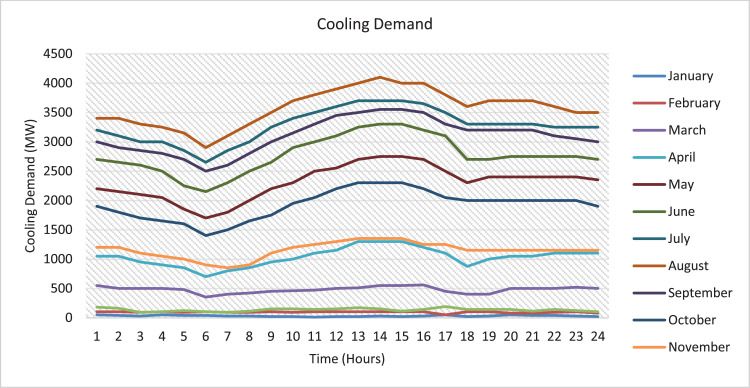
Monthly cooling demand per day for State of Qatar.

**Table 1:** Data collected on absorption chiller component.

Fixed cost ($)	Capacity (kW)	Coefficient of performance
8120	35	0.6
16,704,000	12,000	0.8
2,784,000	6000	0.75
5,568,000	12,000	0.75
5,846,400	12,600	0.75
8,352,000	18,000	0.75
153,336	150	0.7
116,999	50	0.7
128,599	100	0.7
145,999	200	0.7
151,799	250	0.7
163,398	300	0.7
174,998	350	0.7
184,990	400	0.7
198,198	450	0.7
600,000	176	0.7
2,024,000	1547	0.74
4,752,000	4642	0.79
1,980,000	1161	1.42
5,808,000	4642	1.42
2,178,000	1161	1.35
4,000,000	3517	1.38
124,120	233	1.36
229,680	582	1.36
283,040	872	1.36
338,720	1163	1.36
399,040	1454	1.36
443,120	1745	1.36
559,120	2326	1.36
650,760	2908	1.36
737,760	3489	1.36
892,040	4652	1.36
1,053,280	5830	1.36
1,195,960	7000	1.36
1,512,640	9304	1.36
3,493,920	24,000	1.36
5,240,880	36,000	1.36
6,987,840	48,000	1.36
8,734,800	60,000	1.36
1,746,960	12,000	1.36
80,000	100	0.5
2,150,000	5000	1.3
1,228,000	2000	1.2
737,000	1000	1.1
460,500	500	0.8
2,568,031.2	17,640	1.36

**Table 2:** Data collected on solar collectors component.

Solar collector type	Fixed cost ($/m2)	Efficiency
Flat plate and evacuated tube	162$/m2	0.70
Flat collector	300 $/m2	0.75
	500 $/m2	0.75
	700 $/m2	0.75
	900 $/m2	0.75
	1100 $/m2	0.75
	34.19 - 56.98 $/m2	–
	102.56 - 170.93 $/m2	–
	650 $/m2	0.40
	429.61 $/m2	0.40
	859.23 $/ m2	0.40
	1287.70 $/m2	0.40
	533 $/m2	0.21
	505 $/m2	–
	159 $/m2	–
	339 $/m2	0.38
	360 $/m2	0.43
	333.33 $/m2	0.45
	346 $/m2	0.36
	310 $/m2	0.35
	827.32 $/m2	0.327
	747.55 $/m2	0.268
	708.80 $/m2	0.212
	1220.46 $/m2	0.316
	731.59 $/m2	0.371
	711.08 $/m2	0.356
	920.76 $/m2	0.345
	589.15 $/m2	0.346
	1125 $/m2	0.70
Vacuum tube	847 $/m2	0.49
	621 $/m2	0.36
Evacuated tube	1154$/m2	0.44
	858 $/m2	0.63
	827$/m2	0.54
	576 $/m2	0.39
	815 $/m2	0.52
	1148 $/m2	0.57
	740 $/m2	0.42
	2000$/m2	–
	1095 $/m2	–

**Table 3:** Data collected on chilled & hot TES component.

Thermal energy storage type	Capacity (kWh)	Fixed cost ($)
TTES	1,230,898	360,294
	8,616,289	2,522,059
	1,195,730	350,000
	8,370,110	2,450,000
	2,285,954	455,000
	2,180,449	434,000
	3,165,168	630,000
	6,330,335	1,260,000
	18,991,005	3,780,000
	703,371	500,000
	50	580
	14	488
	4	1069
	375,000	495,000
	938	237,600
	1278	308,880
	1,440,000	1,900,800
	8,640,000	11,404,800
	855,000	1,128,600
	156,900	740,305
	261,621	1,105,887
	600,966	1,398,353
PTES	12,000,000	4,752,000
	4,500,000	1,782,000
	3,600,000	1,425,600
	7,200,000	2,851,200
	84,000	33,264
	270,000	106,920
	63,000	24,948
	75,663	29,963
	126,000	49,896
	90,000	35,640
	58,106	462,691
	40,758	196,500
	307,246	726,595
	579,710	913,956
	4,330,985	3,513,021
BTES	285,000	150,480
	562,000	297,000
	504,855	266,563
	900,000	475,200
	949,500	501,336
	140,250	73,920
	290,322	514,100
	261,627	257,050
	619,008	855,692
Hot water tank	576,000	5,220,000
	2556	617,760
	1876	475,200
	2814	712,800
	500	58,000
	750	87,000
	1000	116,000
	1250	145,000
	1500	174,000
	1750	203,000
	2000	232,000
	2250	261,000
	2500	290,000
	17,482	163,369
	34,865	311,888
Chilled water tank	998,786	626,600
	2,707,977	913,000

**Table 4:** Data collected on auxiliary boiler component.

Auxiliary boiler type	Fixed cost ($)	New capacity (kW)	Efficiency
Gas boiler	4,640,000	50,000	0.55
	32,450	234	0.85
Oil boiler	4,640,000	50,000	0.8
	1,392,000	10,000	0.8
	4,176,000	30,000	0.8
	5,568,000	40,000	0.8
	6,960,000	50,000	0.8
	2,784,000	20,000	0.8
	31,500	234	0.82
	82,064	4104	0.85
	123,096	6156	0.85
	164,128	8208	0.85
	205,160	10,260	0.85
	246,192	12,312	0.85
	125,000	7000	0.85
	254,398,4	12,722	0.85
	318,750	17,850	0.85
Liquid fuel boiler	2,250,000	60	0.6
	4,230,000	120	0.6
	9,900,000	300	0.6
	18,900,000	600	0.65
	36,900,000	1200	0.65
	74,250,000	3000	0.7
	148,500,000	6000	0.7
	222,750,000	9000	0.7
	297,000,000	12,000	0.7
Coal boiler	2,250,000	60	0.5
	4,230,000	120	0.5
	9,900,000	300	0.5
	18,900,000	600	0.55
	36,900,000	1200	0.55
	74,250,000	3000	0.6
	41,032	2052	0.85
	182,438	733	0.8
	1,459,504	5861	0.8
	103,010	733	0.8
	486,156	440	0.8
	101,278	733	0.8
	180,674	440	0.8
	95,125	733	0.8
	88,475	586	0.8
Electric boilers	7047	25	0.96
	24,000	100	0.96
	57,000	250	1
	1,328,100	5875	1
	11,750	165	0.98

## 2 Experimental design, materials, and methods

The method used to acquire the collected data in this paper varies between using commercial websites, governmental websites, journal papers, real-life case studies and governmental officials. A vast number of commercial websites, journal papers, and real-life case studies of different countries (i.e., Europe, the United States of America and Qatar) are visited to collect the required information and data on the parameters. Different keywords related to the area of research are used in the research engine during data collection. The raw data collected on a certain parameter had usually different units as they gathered from websites of different countries, so they are filtered to enhance their quality. The filtration process included unifying all measurement units such as currency and power conversions to make all the collected data consistent and then they are included in the below tables. This applies to the data collected on parameters related to the system components which are absorption chiller, solar collectors, auxiliary boiler, chilled and hot water TES tank. However, data of other parameters which are variable costs of producing and storing hot and chilled water, annual hourly global solar radiation and annual hourly cooling demand, the data are collected from other sources. The data of the variable costs of producing and storing hot and chilled water parameters are obtained directly from a governmental website which is Kahramaa's - the water and electricity service provider at Qatar- website. While data of the annual hourly global solar radiation parameter are obtained from Kahramaa's database through a governmental official. The obtained raw data had solar global radiation values from December 2014 to December 2016 for the state of Qatar. However, the data was filtered and only the data related to the year 2016 was extracted and used in the research to match the annual hourly cooling demand data. The data of annual hourly cooling demand is derived using a specific approach explained thoroughly below. The approach uses temperature as an input, so the temperature of the year 2016 for Qatar is used, along with other inputs. The process of collecting data for each parameter of the solar assisted district cooling system is explained below.

### 2.1 Absorption chiller component

The data collected on the Absorption Chiller component includes the following parameters, fixed investment cost of installing a chiller ($), its capacity (kW) and coefficient of performance (COP). The data are collected from commercial websites [2]. Different keywords related to the area of research are used in the research engine during data collection. The collected data are filtered and refined, it means that all the data have consistent units. The number of collected inputs is 46. Table 1 shows the data collected on the absorption chiller component.

### 2.2 Solar collectors component

The data collected on the Solar Collector component includes the following parameters, fixed cost of installing a unit area ($/m^2^), and efficiency of a solar collector. The data are collected from different sources such as real-life case studies and commercial websites [3], [4], [5], [6]. There are 40 inputs. Data are collected on different types of solar collectors which are flat plate, evacuated tube, vacuum tube, and evacuated tube. Different keywords related to the area of research are used in the research engine during data collection. The collected data are filtered and refined, it means that all the inputs have consistent units. Table 2 shows the data collected on the solar collector component.

### 2.3 The chilled and hot water thermal energy storage tank component

The collected data on Thermal Energy Storage (TES) component includes the following parameters, fixed cost of installing chilled water and a hot TES tank ($), and their capacity (kWh). The data are collected from different sources [7], [8], [9]. Most of the collected data are obtained from real-life case studies from all around the world. There are different types of TES that can be used for commercial aspects such as Water Tank Thermal Energy Storage (TTES), Pit Thermal Energy Storage (PTES), and Borehole Thermal Energy Storage (BTES). These types differ in the way they function, installation and duration of storing the heat of the water in the tank (Inter day, seasonal, etc.). The data are collected on different types of TES and different keywords related to the area of research are used in the research engine during data collection. The collected data are filtered and refined, it means that all the inputs have consistent units. The number of collected inputs for hot and chilled water thermal energy storage tank is 63 inputs. Table 3 shows the data collected on the chilled and hot water TES component.

### 2.4 Auxiliary boiler component

The collected data on the auxiliary boiler component includes the following parameters, fixed cost of installing boiler ($), it is capacity (kW) and efficiency. The data are collected from different sources such as real-life case studies and commercial websites [10], [11], [12], [13], [14]. The data are collected on different types of boiler such as oil, gas, and electric boiler. Different keywords related to the area of research are used in the research engine during data collection. The collected data are filtered and refined, it means that all the inputs have consistent units. The number of collected inputs is 46. Table 4 shows the data collected on the auxiliary boiler component.

### 2.5 Variable cost of producing and storing chilled and hot water

The variable cost of producing or storing chilled or hot water at TES is related to the cost of electricity consumption. In Qatar, the cost of electricity is constant throughout the year, so it doesn't vary. Hence, the cost of electricity consumption is obtained directly from the electricity and water service provider in Qatar, which is Kahramaa's website [15]. The variable costs of producing a unit of chilled water from a chiller or a unit of hot water from an auxiliary boiler ($/kW) will be the same as the cost of electricity consumption. Moreover, the variable cost of storing a unit of chilled water at chilled TES or storing a unit of hot water at hot TES ($/kWh) will be the same as the cost of electricity consumption. According to Kahramaa's website [15], the cost of electricity consumption for the commercial industry is 0.055 $/kWh.

### 2.6 Annual hourly global solar radiation

The data required for global solar radiation (W/m^2^) is the annual hourly global solar radiation for the state of Qatar. It is collected from the government sector Kahramaa's database -the water and electricity service provider - at Qatar. Hence, the collected data represents the state of Qatar- Doha. The raw data obtained from the database, through a governmental official, had solar radiation values from December 2014 to December 2016. However, the data was filtered and only the data related to the 2016 year was extracted and used in the research. As an example, the global solar radiation of August is shown in Fig. 1, the complete data and figures are included in the repository. The figures and data indicate that the global solar radiation is obtained during the daytime periods.

### 2.7 Annual hourly cooling demand

**Fig. 3:**
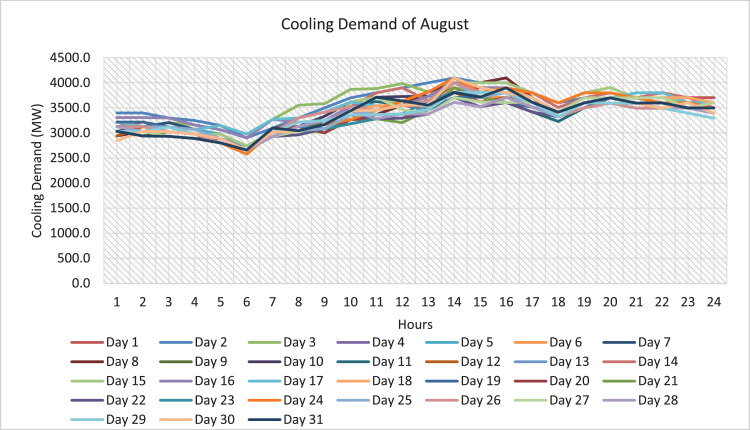
Monthly cooling demand of August.

The annual hourly cooling demands for Qatar state are collected over 8784 h/ year. However, the only cooling demand data available for Qatar is the hourly cooling demand for only a day in the month for the year 2016. They are obtained from a graph included in Quantifying the Cost of Cooling in Qatar [16] as shown in Fig. 3. However, the required data for the cooling demand is the annual hourly cooling demand (i.e., 8784 h/ year). In order to find the cooling demands for the other days in the month, the average temperature for each day in the month is calculated and the day with the highest average temperature in the month is assigned to the cooling demand which is already given in the graph. This day is considered to be a reference day where the cooling demand for the other days is calculated based on this day. So, for the rest of the days in the month, a ratio of the hourly temperature of the day -the day to find the cooling demand for- to the hourly temperature of the reference day multiplied by the cooling demand of that hour of the reference day. By doing so, the required data (i.e., annual hourly cooling demand) are derived from the online available raw data.

$$\begin{array}{ccc} \text{Cooling}\;\text{Demand}\;\text{for}\;a\;\text{day}\;i\;\text{at}\;\text{an}\;\text{hour}\;j & = & \frac{\text{Temperature}\;\text{of}\;\text{day}\;i\;\text{at}\;\text{an}\;\text{hour}\;j}{\text{Temperature}\;\text{of}\;\text{reference}\;\text{day}\;\text{at}\;\text{an}\;\text{hour}\;j}\\ & & \times \;\text{Cooling}\;\text{demand}\;\text{of}\;\text{reference}\;\text{day}\;\text{at}\;\text{an}\;\text{hour}\;j \end{array}$$

The hourly temperature of Qatar is obtained from [17] and these temperatures correspond to the year 2016 to ensure that it is consistent with the data of global solar radiation and cooling demand as they represent the year 2016. The complete figures and data of hourly cooling demands for each day in the month for the 2016 year are included in the repository. Fig. 3 shows the annual hourly cooling demand of August as an example. The pattern is the same as the cooling demand obtained from Quantifying the Cost of Cooling in Qatar [16].
